# The cross-sectional associations between sense of coherence and diabetic microvascular complications, glycaemic control, and patients' conceptions of type 1 diabetes

**DOI:** 10.1186/1477-7525-8-142

**Published:** 2010-11-29

**Authors:** Aila J Ahola, Markku Saraheimo, Carol Forsblom, Kustaa Hietala, Per-Henrik Groop

**Affiliations:** 1 Folkhälsan Institute of Genetics, Folkhälsan Research Center, Biomedicum Helsinki, Finland; 2 Department of Medicine, Division of Nephrology, Helsinki University Hospital, Helsinki, Finland; 3 Division of Nutrition, Department of Food and Environmental Sciences, University of Helsinki, Finland

## Abstract

**Background:**

Sense of coherence (SOC) has been associated with various self-care behaviours in the general population. As the management of type 1 diabetes heavily relies on self-management, the SOC concept could also prove important in this population. This paper is a report of a study conducted among patients with type 1 diabetes to assess the associations between SOC and glycaemic control, microvascular complications, and patients' conceptions of their disease.

**Methods:**

Altogether 1,264 adult patients (45% men, age range 18-82 years) with type 1 diabetes participated in this cross-sectional study. SOC was evaluated using a 13-item SOC questionnaire. Standardized assays were used to determine HbA_1c_. Nephropathy status was based on albumin excretion rate and retinal laser-treatment was used as an indication of severe retinopathy. Patients' subjective conceptions of diabetes were studied using a questionnaire.

**Results:**

Higher SOC scores, reflecting stronger SOC, were associated with lower HbA_1c _values. Strong SOC was independently associated with reaching the HbA_1c _level <7.5%. Adjusting for diabetes duration, age at onset, socioeconomic status and HbA_1c_, weak SOC was associated with the presence of nephropathy among men, but not women. No associations were observed between SOC and severe retinopathy. Four dimensions describing patients' conceptions of HbA_1c_, complications, diabetes control and hypoglycaemia were formed from the diabetes questionnaire. Weak SOC was independently associated with worse subjective conceptions in the dimensions of HbA_1c _and hypoglycaemia. Furthermore among men, an association between weak SOC and the complications factor was observed.

**Conclusion:**

Interventions to improve patients' SOC, if available, could improve patients' metabolic control and therefore also reduce the incidence of diabetic complications.

## Introduction

Type 1 diabetes is a chronic disease that is frequently associated with severe vascular complications [1]. Importantly, diabetic complications account for the major morbidity and mortality associated with the disease [2]. Therefore prevention of these complications, by means of strict glycaemic control, is of major importance in the management of type 1 diabetes [3]. Conscientious daily self-care practices, that include frequent blood glucose monitoring and meticulous meal planning, are strongly emphasized [4,5], but despite these meeting the treatment goals has shown to be fairly difficult [6]. Therefore, identification of factors that contribute to the improved diabetes management would be of specific importance.

The theoretical model of the sense of coherence (SOC) has been associated with various self-care behaviours including cigarette smoking, physical activity, food selection and oral health-related behaviours [7-9]. Antonovsky identified and advocated the use of SOC as a central part of his salutogenic approach that explains why some individuals stay healthy despite of encountering major stressors, while others do not [10]. In this model, health and disease form a continuum from "ease" to "disease", rather than are seen as a dichotomous variable. Whether an individual moves from one end to another, along this continuum, depends on the encountered stressors and the set of coping resources available. According to Antonovsky, comprehensibility, manageability and meaningfulness are the three components that constitute the SOC. These components express the extent to which one has a pervasive feeling of confidence that the confronted stimuli are structured and predictable (comprehensible), worthy of engagement (meaningful), and that an individual has sufficient resources to meet the demands of life (manageable). The stronger the SOC, the more likely an individual is able to select appropriate coping strategies and therefore to move towards the "ease" end of the continuum.

Considering that the management of type 1 diabetes relies heavily on self-management practices, the SOC construct could also prove valuable in this patient group. Currently the SOC is, however, rather unexplored among patients with type 1 diabetes and the available data are inconsistent. While in two small studies conducted among patients with insulin-dependent diabetes, SOC was not associated with the metabolic control [11,12], in a mixed population consisting of patients with type 1 and type 2 diabetes, strong SOC was associated with better glycaemic control [13].

Due to the limited and conflicting evidence obtained thus far, we aimed to investigate whether weak SOC is associated with glycaemic control and microvascular complications among patients with type 1 diabetes. Additionally, the association between SOC and patients' subjective conceptions of the disease was investigated. We hypothesised that weak SOC is associated with poor metabolic control and the presence of microvascular complications. Furthermore we expected weak SOC to be reflected in more negative self-reports of diabetes and its management.

## Methods

Cross-sectional data from a total of 1,264 patients with type 1 diabetes participating in the Finnish Diabetic Nephropathy Study (FinnDiane) were included. Since its launch in 1997, data from more than 4,800 patients have been collected in the FinnDiane Study. However, the collection of SOC data did not start until September 2003. In the present study, all patients providing SOC data by April 2010 were included. The study protocol was approved by the Ethics Committee of the Hospital District of Helsinki and Uusimaa, and patients gave written informed consent prior to participation.

During the visits, patients were provided with questionnaires to be completed at home. First, Antonovsky's 13-item SOC questionnaire was applied [10]. The questionnaire has shown to be reliable, valid and cross culturally applicable [14]. On a seven point Likert-type scale, patients select a reply for each question between two extremes (i.e. very often and never). After a reverse scoring for questions 1, 2, 3, 7, and 10, all items were summed to obtain the SOC score. Patients that did not provide answers to all questions were excluded (n = 47). In the questionnaire, potential scores range between 13 and 91; the higher the score, the stronger the SOC. The ordinal SOC score was used as a continuous variable and patients were additionally divided into quartiles based on their SOC score, as previously seen [8]. The lowest quartile was considered to have weak SOC, and was compared to the remaining patients.

Second, in a structured questionnaire, patients were asked to provide answers to various diabetes specific questions (Additional file 1, Diabetes questionnaire). The questionnaire was designed for the purpose of this study by a panel of experienced physicians who actively participate in the clinical work. A special emphasis was placed on items that are of clinical relevance in patients' daily lives. Amongst others, questions inquiring patients' satisfaction with their current HbA_1c _and insulin regimen were included. Patients were instructed to select the most appropriate answer from the predetermined alternatives.

HbA_1c _was determined locally by standardized assays. Serum lipid and lipoprotein concentrations were measured as previously described [15]. Following a 10-minute rest, blood pressure was measured twice with two minutes intervals in the sitting position. Blood pressure was calculated as a mean of these measurements. Patients' height and weight were measured in light clothing and body mass index (BMI) was calculated (kg/m^2^). The assessment of renal status was based on urinary albumin excretion rate (AER) in at least two out of three timed 24-h or overnight urine collections. Patients were classified according to the following criteria: normal albumin excretion rate (AER <20 μg/min or <30 mg/24 h), microalbuminuria (AER ≥20 and <200 μg/min or ≥30 and <300 mg/24 h), macroalbuminuria (AER ≥200 μg/min or ≥300 mg/24 h), or end-stage renal disease (ESRD) (undergoing dialysis or having had a kidney transplant). Diabetic nephropathy was defined as macroalbuminuria or ESRD. Data on retinopathy were obtained from medical records and retinal laser-treatment was used as an indication of severe retinopathy [16]. Self-reported data for smoking and social class (grouped as unskilled blue collar, n = 152; skilled blue collar, n = 390; lower white collar, n = 227; upper white collar, n = 182; farmers, n = 4; and others, n = 46) were collected. Unskilled blue collar workers were classified as having a low socioeconomic status (SES).

### Statistical analyses

Descriptive statistics are reported as percentages for categorical data, mean ± SD for normally distributed continuous data, and median (interquartile range) for non-normally distributed continuous data. Group comparisons were performed using the Chi-squared test, independent-sample t-test, and Mann Whitney U-test, as appropriate. Spearman rank order correlation coefficient was calculated to study the relationship between SOC score and HbA_1c_, as they were not normally distributed. Logistic regression analyses were used to explore the independent associations between weak SOC and complications and glycaemic control. Exploratory factor analysis (maximal likelihood and varimax rotation) was used to identify underlying constructs within the diabetes questionnaire. The number of factors identified was based on eigenvalues >1.0. Items were considered to load highly if they had a factor loading |≥0.20| with a particular factor. The factor score was the sum of the scores for all items associated with a given factor multiplied by its corresponding factor loading. These scores were used as dependent variables in analyses. The questionnaire's reliability was assessed calculating Cronbach's α, and α > 0.60 was deemed acceptable. Factorial analysis of variance was used to study the associations between SOC status and the measured HbA_1c _and the factors formed in the factor analysis. In the analyses, gender interactions were evaluated and, when applicable, separate analyses for men and women were performed. All data were analyzed using SPSS 17.0 for Windows (SPSS, Chicago, IL).

## Results

A total of 61% of the patients returned the questionnaires. The ones returning the questionnaire were older (mean age 45 ± 12 years vs. 42 ± 13 years, *p *< 0.001) and had longer diabetes duration (28 ± 13 vs. 24 ± 13, *p *< 0.001) compared to those not returning the questionnaire. The proportion of men was higher among the non-responders (55% vs. 45%, *p *< 0.001). Moreover, the non-responders were more frequently smokers (24% vs. 19%, *p *= 0.008) and had more frequently low socioeconomic status (20% vs. 15% *p *= 0.013). No differences were observed in any of the laboratory values, blood pressure, BMI, and in the nephropathy or retinopathy status.

Data from a total of 1,264 patients (45% men) are included (Table 1). Mean age was 45 ± 12 years and duration of diabetes 28 ± 13 years. Based on the lowest quartile, a cut-off point of <63 was set for the weak SOC, while the median SOC score in the population was 73. Compared to men, women had lower median SOC scores [74 (65 - 81) vs. 72 (61 - 80), *p *= 0.003]. Moreover, the prevalence of weak SOC was higher among women (28% vs. 20%, *p *= 0.001).

**Table 1:** Description of the study population

	**Weak SOC**^**a**^	Strong SOC	*p*
	n = 311 (25%)	n = 953 (75%)	
Men, n (%)	113 (36)	453 (48)	0.001
Age, years	44 ± 12	45 ± 12	0.266
Diabetes duration, years	29 (19 - 38)	28 (18 - 37)	0.270
Current smoking, %	21	18	0.273
Low SES ^b^, %	15	15	1.000
HbA_1c_, %	8.1 (7.5 - 9.0)	8.0 (7.2 - 8.7)	0.004
Total cholesterol, mmol/l	4.6 (4.0 - 5.1)	4.4 (4.0 - 5.1)	0.352
HDL cholesterol, mmol/l	1.8 (1.4 - 2.1)	1.7 (1.4 - 2.0)	0.551
Triglycerides, mmol/l	0.9 (0.6 - 1.3)	0.9 (0.7 - 1.3)	0.842
BMI, kg/m^2^	25 (23 - 28)	25 (23 - 28)	0.625
Systolic blood pressure, mmHg	135 (124 - 151)	137 (125 - 151)	0.204
Diastolic blood pressure, mmHg	80 (71 - 85)	79 (72 - 85)	0.941

Data are shown as frequency (%) for categorical variables, mean ± SD for continuous normally distributed variables, and median (interquartile range) for continuous non-normally distributed variables. ^a ^SOC = sense of coherence; values <63 signify weak SOC, ^b ^Low socioeconomic status (unskilled blue collar workers).

### SOC and metabolic control

The SOC score was negatively associated with the measured HbA_1c _(r = -0.091, *p *= 0.002). The median HbA_1c _was higher among those with weak SOC [8.1 (7.5 - 9.0) vs. 8.0 (7.2 - 8.7), *p *= 0.004]. Moreover, patients with strong SOC more frequently achieved an HbA_1c _level below 7.5% (32% vs. 24%, *p *= 0.016).

### SOC, nephropathy and retinopathy

Nephropathy status was evaluated in 1,118 patients. A total of 276 (25%) patients had nephropathy judged by the presence of either macroalbuminuria or ESRD. Weak SOC was observed in 29% and 24% of the patients with and without nephropathy (*p *= 0.110). No difference in the median SOC score was observed between patients with and without nephropathy [71 (59 - 79) vs. 73 (63 - 80), *p *= 0.089]. Data on retinopathy were available from 1,239 patients. Altogether 461 (37%) patients had severe retinopathy. Weak SOC was an equally common finding among patients with and without severe retinopathy (27% vs. 23%, *p *= 0.134, respectively), and the respective median SOC score was no different [72 (61 - 80) vs. 73 (63 - 80), *p *= 0.351].

Unlike among women, weak SOC was independently associated with nephropathy in men when adjusted for diabetes duration, age at onset, socioeconomic status and HbA_1c _(Table 2). No association was observed between weak SOC and retinopathy in either sex. After adjustments with gender, diabetes duration, age at onset, socioeconomic status and HbA_1c_, weak SOC was associated with the measured HbA_1c _values above 7.5%.

**Table 2:** The association between weak sense of coherence and nephropathy, severe retinopathy and HbA1c level.

	Nephropathy		Severe retinopathy		**HbA**_**1c **_**≥7.5%**
	Men	Women	Men	Women	Men and women combined
Model 1					
Weak SOC	**1.97 (1.26 - 3.08)**	1.07 (0.69 - 1.66)	**1.53 (1.01 - 2.33)**	1.15 (0.81 - 1.63)	**1.44 (1.06 - 1.97)**
Gender, 1 = male	NA	NA	NA	NA	0.84 (0.65 - 1.08)
Model 2					
Weak SOC	**1.97 (1.15 - 3.05)**	1.03 (0.65 - 1.62)	1.39 (0.85 - 2.27)	1.20 (0.80 - 1.81)	**1.43 (1.04 - 1.95)**
Gender, 1 = male	NA	NA	NA	NA	0.83 (0.64 - 1.08)
Diabetes duration, years	**1.07 (1.05 - 1.09)**	**1.04 (1.02 - 1.06)**	**1.11 (1.09 - 1.13)**	**1.10 (1.08 - 1.12)**	0.99 (0.98 - 1.00)
Age at onset, years	0.99 (0.97 - 1.01)	**0.95 (0.92 - 0.98)**	0.99 (0.97 - 1.01)	**0.94 (0.92 - 0.97)**	0.99 (0.98 - 1.01)
Model 3					
Weak SOC	**2.11 (1.13 - 3.95)**	0.80 (0.44 - 1.45)	1.15 (0.63 - 2.11)	1.23 (0.75 - 2.03)	**1.52 (1.07 - 2.17)**
Gender, 1 = male	NA	NA	NA	NA	0.86 (0.64 - 1.15)
Diabetes duration, years	**1.10 (1.07 - 1.13)**	**1.04 (1.01 - 1.06)**	**1.12 (1.09 - 1.15)**	**1.11 (1.08 - 1.13)**	0.99 (0.98 - 1.01)
Age at onset, years	1.00 (0.94 - 1.03)	**0.95 (0.92 - 0.99)**	1.00 (0.97 - 1.03)	**0.95 (0.92 - 0.98)**	1.00 (0.98 - 1.01)
Low SES, 1 = yes	1.50 (0.76 - 2.97)	1.96 (0.95 - 4.05)	1.14 (0.59 - 2.21)	1.39 (0.70 - 2.75)	0.81 (0.54 - 1.23)
HbA_1c_	**1.37 (1.12 - 1.69)**	**1.32 (1.08 - 1.61)**	**1.36 (1.11 - 1.67)**	1.17 (0.97 - 1.40)	NA

Data are presented as odds ratio (95% confidence interval). Weak SOC, weak sense of coherence; Low SES, low socioeconomic status (unskilled blue collar workers); NA, not applicable. Logistic regression analysis. Significant results are highlighted in bold.

### Factor analysis and reliability of the diabetes questionnaire

Four factors consisting of questions with a high degree of intercorrelation were formed. The first factor called "conceptions of HbA_1c_" (eigenvalue 2.71, explained variance 19%), consisted of questions regarding patient's recollection of the last measured HbA_1c _value, patient's perception of whether that value was at a good, satisfactory or high level, and whether patient was satisfied with that HbA_1c _level. The second factor was named "complications" (eigenvalue 2.17, explained variance 15%) and included questions about the numbers of doctors' and nurses' appointments during the past year due to non-diabetes related reasons, the presence of other chronic illnesses and patient's perception of how much diabetes-related complications cause disturbance. Questions on the frequencies of diabetes-related doctors' and nurses' visits during the past year formed the "diabetes control" factor (eigenvalue 1.42, explained variance 10%). Finally, the factor "hypoglycaemia" (eigenvalue 1.18, explained variance 8%) was formed of questions on patient's perceived fear of hypoglycaemia, satisfaction with the current insulin regimen, the frequency of experiencing hypoglycaemic episodes, patient's perception of how much diabetes *per se *or its treatment disturbs everyday life and patient's perception of how much diabetes-related complications cause disturbance. In all the four factors, higher factor scores denote less favourable situation, e.g. higher self-reported HbA_1c_, perception of it being at a high level and lower satisfaction with the current HbA_1c _level in the conceptions of HbA_1c _-factor. The reliability analysis of the questionnaire gave a Cronbach's alpha value of 0.625.

### SOC and patients' conceptions of diabetes

The SOC score was negatively associated with three factor scores; conceptions of HbA_1c _(r = -0.116, *p *< 0.001), complications (r = -0.163, *p *< 0.001), and hypoglycaemia (r = -0.342, *p *< 0.001), suggesting that patients with weak SOC have less favourable conceptions of these diabetes related dimensions.

Using a factorial analysis of variance, weak SOC was associated with the measured HbA_1c _when adjusted for gender, diabetes duration, age at onset, and SES (Table 3). Of the four factors formed, weak SOC was independently associated with the conceptions of HbA_1c _and hypoglycaemia. Furthermore among men, weak SOC was associated with the complications factor.

**Table 3:** The association between weak sense of coherence, HbA1c and four dimensions of the diabetes questionnaire.

	Model 1			Model 2		
	***F*(df**_**M**_**, df**_**R**_**)**	*p*	**η**^**2**^	***F*(df**_**M**_**, df**_**R**_**)**	*p*	**η**^**2**^
Measured HbA_1c_	*F*(1, 1109) = 8.80	0.003	0.008	*F*(1, 865) = 6.03	0.014	0.007
Conceptions of HbA_1c_	*F*(1, 1241) = 14.14	< 0.001	0.011	*F*(1, 980) = 9.34	0.002	0.009
Complications						
Men	*F*(1, 552) = 27.81	< 0.001	0.048	*F*(1, 429) = 23.94	< 0.001	0.053
Women	*F*(1, 687) = 3.55	0.060	0.005	*F*(1, 548) = 1.99	0.159	0.004
Diabetes control	*F*(1, 1241) = 2.97	0.085	0.002	*F*(1, 980) = 1.61	0.205	0.002
Hypoglycaemia	*F*(1, 1241) = 114.76	< 0.001	0.085	*F*(1, 980) = 80.00	< 0.001	0.075

Model 1 is adjusted for gender (except for complications), diabetes duration and age at onset of diabetes; Model 2 is further adjusted for socio-economic status. Factorial analysis of variance

## Discussion

According to Antonovsky, SOC is established during the childhood and adolescence and having developed, he considered it fairly stable quality throughout the adulthood [10]. Importantly, SOC has been associated with health-promoting behaviour, such as more prudent dietary habits and a reduced likelihood of being a smoker and physically inactive [7]. As the successful management of type 1 is heavily dependent on patient's self-management, we hypothesized that SOC could also play an important role among these patients. Indeed, in the current study we observed that weak SOC was independently associated with poorer glycaemic control and with the presence of nephropathy among men. Our results, therefore, give some support to the SOC theory that suggests that patients with strong SOC might be better able to use any resources available to enhance their well-being.

In the current study, weak SOC was not associated with severe retinopathy in either sex. However, among men, weak SOC was associated with nephropathy even after adjustment with more established risk factors, such as diabetes duration and HbA_1c_. The reason for the observed gender difference in the association between SOC and nephropathy is not known. A potential contributing factor might, however, be the fact that compared to women, men were more frequently smokers. Further adjustment with smoking did not, however, alter the results (data not shown). The results are intriguing also because, as seen also in some other studies [7,12], women tend to have lower SOC scores and thus have a higher prevalence of weak SOC. Our results suggest that, despite lower SOC scores, women as opposed to men might be more able to engage in health promoting behaviours. This also raises the question whether different cut-off values for men and women should be used when defining weak SOC.

Previously Richardson et al. observed a lower mean SOC score among patients with two or more complications as opposed to those with less complications [12]. Except for this report, studies on the association between SOC and diabetic complications are scarce. However, a number of study reports on SOC and metabolic control are available. Two of these studies, conducted among patients with type 2 diabetes, found no direct association between SOC and HbA_1c _[17,18]. Two further studies among patients with insulin-dependent diabetes also concluded that SOC and glycaemic control are not correlated [11,12]. Only evidence, to our knowledge, relating SOC to glycaemic control comes from Cohen and Kanter [13]. According to them, however, the relationship was not direct but was mediated via adherence to self-care behaviours and psychological distress.

In clinical practice, monitoring glycaemic control and any signs of vascular complications are of major importance. Beyond these hard end points, however, patients' subjective conceptions of their disease are also important. Indeed, some of the major observations in the studies of SOC and diabetes have dealt with acceptance of the disease and the subjective assessment of health state. Based on the results from these previous studies, patients with type 1 diabetes who have higher SOC scores also have a higher degree of disease acceptance [12] and also less problems in relation to the environment, tedium and well being [11].

In the current study, patients' conceptions of diabetes were studied using a questionnaire from which, using factor analysis, four dimensions were identified. Of these dimensions, weak SOC was associated with worse conceptions of HbA_1c _meaning that their latest self-reported HbA_1c _measurement was higher, they more frequently considered their HbA_1c _being at poor level, and they less frequently were satisfied with their HbA_1c _level. Of the other dimensions, weak SOC was also associated with the hypoglycaemia-factor, that amongst other items contained questions about fear of hypoglycaemia and satisfaction with the current insulin regimen. Furthermore, men also showed an association between weak SOC and the complications-factor. These results are in line with previous studies that reported that strong SOC was associated with better self-assessed health [18] and lower fear of hypoglycaemia [17]. In diabetes, fear of hypoglycaemia is specifically important as it is not only a subjective nuisance, but it may also affect patients' insulin regimen and eating habits. Indeed, it has been suggested that fear of hypoglycaemia might promote non-adherence behaviour in order to avoid hypoglycaemic episodes [19]. Moreover, in a recent review, a significant negative impact between fear of hypoglycaemia and diabetes management and metabolic control was shown [20].

Our study has both strengths and limitations. A major strength is the large number of patients included in this study. Additionally, the diagnoses of diabetes and complications were based on a physician's evaluation rather than on self-reported data, reducing the possibility of misclassification. The major limitation is the cross-sectional nature of the study design that limits the determination of temporal relationships. Although Antonovsky suggested that, after having established, SOC is a fairly stable phenomenon, we do not know whether SOC is indeed a primary feature or rather a consequence of glycaemic control or diabetic complications. There are currently some evidence to suggest that SOC could be improved using psychological intervention [21,22]. Therefore longitudinal associations between SOC, and glycaemic control and diabetic complications will need to be addressed in the future analyses. This study may further be limited by the fact that while SOC was studied using a validated questionnaire, patients' perceptions of diabetes were studied with a non-validated questionnaire. When formulating the questionnaire, special emphasis was placed on including items that are of clinical relevance in patients' daily lives. Of specific importance is that many of these questions are also addressed in actual clinical work, for which we believe our results may provide a valuable contribution. Another potential limitation is that marital status was not assessed in the current study. It can be speculated that a spouse can also influence the individual's self-care behaviours and therefore have an effect on the outcome measures. Finally, although we consider that the FinnDiane study population is a fairly representative of the Finnish adult patients with type 1 diabetes, some selection bias favouring women and older individuals was observed in the current study population. It is also possible that individuals with weak SOC and those with more severe health problems were under represented, an observation that is likely to attenuate the results.

In conclusion, strong SOC was associated with a better metabolic control among men and women, and the presence of nephropathy among men. Strong SOC was also associated with better patients' conceptions of their disease. Interventions to improve patients' SOC, if available, could improve patients' metabolic control and therefore also reduce the risk of diabetic complications.

## Appendix

The physicians and nurses participating in the enrolment of patients:  

Anjalankoski Health Center: S. Koivula and T. Uggeldahl; Central Finland Central Hospital: T. Fors-lund, A. Halonen, A. Koistinen, P. Koskiaho, M. Laukkanen, J. Saltevo and M. Tiihonen; Central Hospital of Åland Islands: M. Forsen, H. Granlund, A.-C. Jonsson and B. Nyroos; Central Hospital of Kanta-Häme: P. Kinnunen, A. Orvola, T. Salonen and A. Vähänen; Central Hospital of Kymenlaakso: R. Paldanius, M. Riihelä and L. Ryysy; Central Hospital of Länsi-Pohja: H. Laukkanen, P. Nyländen and A. Sademies; Central Ostrobothnian Hospital District: S. Anderson, B. Asplund, U. Byskata, P. Liedes, M. Kuusela and T. Virkkala; City of Espoo Health Center (Espoonlahti): A. Nikkola and E. Ritola; (Tapiola): M. Niska and H. Saarinen; (Viherlaakso): A. Lyytinen; City of Helsinki Health Center (Puistola): H. Kari and T. Simonen; (Suutarila): A. Kaprio, J. Kärkkäinen and B. Rantaeskola; (Töölö): P. Kääriäinen, J. Haaga and A-L. Pietiläinen; City of Hyvinkää Health Center: S. Klemetti, T. Nyandoto, E. Rontu and S. Satuli-Autere; City of Vantaa Health Center (Korso): R. Toivonen and H. Virtanen; (Länsimäki): R. Ahonen, M. Ivaska-Suomela and A. Jauhiainen; (Martinlaakso): M. Laine, T. Pellonpää and R. Puranen; (Myyrmäki): A. Airas, J. Laakso and K. Rautavaara; (Rekola): M. Erola and E. Jatkola; (Tikkurila): R. Lönnblad, A. Malm, J. Mäkelä and E. Rautamo; Heinola Health Center: P. Hentunen and J. Lagerstam; Helsinki University Central Hospital (Department of Medicine, Division of Nephrology): D. Cordin, J. Fagerudd, M. Feodoroff, O. Heikkilä, L. Kyllönen, J. Kytö, K. Pettersson-Fernholm, M. Rosengård-Bärlund, M. Rönnback, L. Thorn and J. Wadén; Herttoniemi Hospital: V. Sipilä; Hospital of Lounais-Häme: T. Kalliomäki, J. Koskelainen, R. Nikkanen, N. Savolainen, H. Sulonen and E. Valtonen; Iisalmi Hospital: E. Toivanen; Jokilaakso Hospital: A. Parta and I. Pirttiniemi; Jorvi Hospital: S. Aranko, S. Ervasti, R. Kauppinen-Mäkelin, A. Kuusisto, T. Leppälä, K. Nikkilä and L. Pekkonen; Jyväskylä Health Center: K. Nuorva and M. Tiihonen; Kainuu Central Hospital: S. Jokelainen, P. Kemppainen, A-M. Mankinen and M. Sankari; Kerava Health Center: H. Stuckey and P. Suominen; Kirkkonummi Health Center: A. Lappalainen, M. Liimatainen and J. Santaholma; Kivelä Hospital: A. Aimolahti and E. Huovinen; Koskela Hospital: V. Ilkka and M. Lehtimäki; Kotka Health Center: E. Pälikkö-Kontinen and A. Vanhanen; Kouvola Health Center: E. Koskinen and T. Siitonen; Kuopio University Hospital: E. Huttunen, R. Ikäheimo, P. Karhapää, P. Kekäläinen, M. Laakso, T. Lakka, E. Lampainen, L. Moilanen, L. Niskanen, U. Tuovinen, I. Vauhkonen and E. Voutilainen; Kuusamo Health Center: T. Kääriäinen and E. Isopoussu; Kuusankoski Hospital: E. Kilkki, I. Koskinen and L. Riihelä; Laakso Hospital, Helsinki: T. Meriläinen, P. Poukka, R. Savolainen and N. Uhlenius; Lahti City Hospital: A. Mäkelä and M. Tanner; Lapland Central Hospital: L. Hyvärinen, S. Severinkangas and T. Tulokas; Lappeenranta Health Center: P. Linkola and I. Pulli; Lohja Hospital: T. Granlund, M. Saari and T. Salonen; Länsi-Uusimaa Hospital: I.-M. Jousmaa and J. Rinne; Loimaa Health Center: A. Mäkelä and P. Eloranta; Malmi Hospital: H. Lanki, S. Moilanen and M. Tilly-Kiesi; Mikkeli Central Hospital: A. Gynther, R. Manninen, P. Nironen, M. Salminen and T. Vänttinen; Mänttä Regional Hospital: I. Pirttiniemi and A-M. Hänninen; North Karelian Hospital: U-M. Henttula, P. Kekäläinen, M. Pietarinen, A. Rissanen and M. Voutilainen; Nurmijärvi Health Center: A. Burgos and K. Urtamo; Oulaskangas Hospital: E. Jokelainen, P.-L. Jylkkä, E. Kaarlela and J. Vuolaspuro; Oulu Health Center: L. Hiltunen, R. Häkkinen and S. Keinänen-Kiukaanniemi; Oulu University Hospital: R. Ikäheimo; Päijät-Häme Central Hospital: H. Haapamäki, A. Helanterä, S. Hämäläinen, V. Ilvesmäki and H. Miettinen; Palokka Health Center: P. Sopanen and L. Welling; Pieksämäki Hospital: V. Javtsenko and M. Tamminen; Pietarsaari Hospital: M-L. Holmbäck, B. Isomaa and L. Sarelin; Pori City Hospital: P. Ahonen, P. Merensalo and K. Sävelä; Porvoo Hospital: M. Kallio, B. Rask and S. Rämö; Raahe Hospital: A. Holma, M. Honkala, A. Tuomivaara and R. Vainionpää; Rauma Hospital: K. Laine, K. Saarinen and T. Salminen; Riihimäki Hospital: P. Aalto, E. Immonen and L. Juurinen; Salo Hospital: A. Alanko, J. Lapinleimu, P. Rautio and M. Virtanen; Satakunta Central Hospital: M. Asola, M. Juhola, P. Kunelius, M.-L. Lahdenmäki, P. Pääkkönen and M. Rautavirta; Savonlinna Central Hospital: T. Pulli, P. Sallinen, M. Taskinen, E. Tolvanen, H. Valtonen and A. Vartia; Seinäjoki Central Hospital: E. Korpi-Hyövälti, T. Latvala and E. Leijala; South Karelia Central Hospital: T. Ensala, E. Hussi, R. Härkönen, U. Nyholm and J. Toivanen; Tampere Health Center: A. Vaden, P. Alarotu, E. Kujansuu, H. Kirkkopelto-Jokinen, M. Helin, S. Gummerus, L. Calonius, T. Niskanen, T. Kaitala and T. Vatanen; Tampere University Hospital: I. Ala-Houhala, T. Kuningas, P. Lampinen, M. Määttä, H. Oksala, T. Oksanen, K. Salonen, H. Tauriainen and S. Tulokas; Tiirismaa Health Center: T. Kivelä, L. Petlin and L. Savolainen; Turku Health Center: I. Hämäläinen, H. Virtamo and M. Vähätalo; Turku University Central Hospital: K. Breitholz, R. Eskola, K. Metsärinne, U. Pietilä, P. Saarinen, R. Tuominen and S. Äyräpää; Vaajakoski Health Center: K. Mäkinen and P. Sopanen; Valkeakoski Regional Hospital: S. Ojanen, E. Valtonen, H. Ylönen, M. Rautiainen and T. Immonen; Vammala Regional Hospital: I. Isomäki, R. Kroneld and M. Tapiolinna-Mäkelä; Vaasa Central Hospital: S. Bergkulla, U. Hautamäki, V.-A. Myllyniemi and I. Rusk.    

## List of abbreviations

SOC: sense of coherence; SES: socioeconomic status; ESRD: end-stage renal disease; AER: albumin excretion rate.

## Competing interests

The authors declare that they have no competing interests.

## Authors' contributions

PHG, MS and CF designed the diabetes questionnaire. KH participated and coordinated the collection of the retinopathy data. AJA performed the statistical analyses and wrote the manuscript. All of the authors reviewed the manuscript prior to submission.

## Supplementary Material

Additional file 1 **Diabetes questionnaire**. Questionnaire on patients' conception of their disease. Click here for file

## References

[B1] Lüscher TF Creager MA Beckman JA Cosentino F Diabetes and vascular disease: pathophysiology, clinical consequences, and medical therapy: Part II Circulation 2003 108 1655 1661 10.1161/01.CIR.0000089189.70578.E2 14517152

[B2] Daneman D Type 1 diabetes Lancet 2006 367 847 858 10.1016/S0140-6736(06)68341-4 16530579

[B3] The Diabetes Control and Complications Trial Research Group The effect of intensive treatment of diabetes on the development and progression of long-term complications in insulin-dependent diabetes mellitus N Engl J Med 1993 329 977 986 10.1056/NEJM199309303291401 8366922

[B4] American Diabetes Association Standards of medical care in diabetes - 2010 Diabetes Care 2010 33 Suppl 1 11 61 15618112

[B5] American Diabetes Association Nutrition recommendations and interventions for diabetes: a position statement of the American Diabetes Association Diabetes Care 2008 31 Suppl 1 61 78 10.2337/dc08-S061 18165339

[B6] Ahola AJ Mäkimattila S Saraheimo M Mikkilä V Forsblom C Freese R Groop PH Many patients with Type 1 diabetes estimate their prandial insulin need inappropriately Journal of Diabetes 2010 2 194 202 10.1111/j.1753-0407.2010.00086.x 20923484

[B7] Wainwright NW Surtees PG Welch AA Luben RN Khaw KT Bingham SA Healthy lifestyle choices: could sense of coherence aid health promotion? J Epidemiol Community Health 2007 61 871 876 10.1136/jech.2006.056275 17873222 PMC2652963

[B8] Lindmark U Stegmayr B Nilsson B Lindahl B Johansson I Food selection associated with sense of coherence in adults Nutr J 2005 4 9 10.1186/1475-2891-4-9 15737236 PMC554973

[B9] Bernabé E Kivimäki M Tsakos G Suominen-Taipale AL Savolainen J Uutela A Sheiham A Watt RG The relationship among sense of coherence, socio-economic status, and oral health-related behaviours among Finnish dentate adults Eur J Oral Sci 2009 117 413 418 10.1111/j.1600-0722.2009.00655.x 19627353

[B10] Antonovsky A Unraveling the Mystery of Health, How People Manage Stress and Stay Well 1987 San Francisco: Jossey-Bass

[B11] Lundman B Norberg A The significance of a sense of coherence for subjective health in persons with insulin-dependent diabetes J Adv Nurs 1993 18 381 386 10.1046/j.1365-2648.1993.18030381.x 8450132

[B12] Richardson A Adner N Nordström G Persons with insulin-dependent diabetes mellitus: acceptance and coping ability J Adv Nurs 2001 33 758 763 10.1046/j.1365-2648.2001.01717.x 11298213

[B13] Cohen M Kanter Y Relation between sense of coherence and glycemic control in type 1 and type 2 diabetes Behav Med 2004 29 175 183 10.3200/BMED.29.4.175-185 15369198

[B14] Eriksson M Lindstrom B Validity of Antonovsky's sense of coherence scale: a systematic review J Epidemiol Community Health 2005 59 460 466 10.1136/jech.2003.018085 15911640 PMC1757043

[B15] Thorn LM Forsblom C Fagerudd J Thomas MC Pettersson-Fernholm K Saraheimo M Wadén J Rönnback M Rosengård-Bärlund M Björkesten CG Taskinen MR Groop PH Metabolic syndrome in type 1 diabetes: association with diabetic nephropathy and glycemic control (the FinnDiane study) Diabetes Care 2005 28 2019 2024 10.2337/diacare.28.8.2019 16043748

[B16] Grassi MA Mazzulla DA Knudtson MD Huang WW Lee KE Klein BE Nicolae DL Klein R Patient self-report of prior laser treatment reliably indicates presence of severe diabetic retinopathy Am J Ophthalmol 2009 147 501 504 10.1016/j.ajo.2008.09.016 19054495 PMC2758026

[B17] Shiu AT Sense of coherence amongst Hong Kong Chinese adults with insulin-treated type 2 diabetes Int J Nurs Stud 2004 41 387 396 10.1016/j.ijnurstu.2003.10.010 15050850

[B18] Sanden-Eriksson B Coping with type-2 diabetes: the role of sense of coherence compared with active management J Adv Nurs 2000 31 1393 1397 10.1046/j.1365-2648.2000.01410.x 10849151

[B19] Cox DJ Irvine A Gonder-Frederick L Nowacek G Butterfield J Fear of hypoglycemia: quantification, validation, and utilization Diabetes Care 1987 10 617 621 10.2337/diacare.10.5.617 3677982

[B20] Wild D von Maltzahn R Brohan E Christensen T Clauson P Gonder-Frederick L A critical review of the literature on fear of hypoglycemia in diabetes: Implications for diabetes management and patient education Patient Educ Couns 2007 68 10 15 10.1016/j.pec.2007.05.003 17582726

[B21] Weissbecker I Salmon P Studts JL Floyd AR Dedert EA Sephton SE Mindfulness-based stress reduction and sense of coherence among women with fibromyalgia J Clin Psychol Settings 2002 9 297 307 10.1023/A:1020786917988

[B22] Delbar V Benor DE Impact of a nursing intervention on cancer patients' ability to cope J Psychosoc Oncol 2001 19 57 75 10.1300/J077v19n02_04

