# Supplementary material for: The cross-sectional associations between sense of coherence and diabetic microvascular complications, glycaemic control, and patients' conceptions of type 1 diabetes
Source: Health Qual Life Outcomes. 2010 Nov 29;8:142. doi: 10.1186/1477-7525-8-142 (PMC3009698; doi:10.1186/1477-7525-8-142)
Supplement: Additional file 1 — Diabetes questionnaire. Questionnaire on patients' conception of their disease. [file 1477-7525-8-142-S1.DOC]

**Diabetes-related questions**

1. How many times, during the preceding year, have you visited a doctor? (please circle the most appropriate alternative)

Due to diabetes 0 1-2 3-4 5-6 7-8 More than 8 times

Due to other reasons 0 1-2 3-4 5-6 7-8 More than 8 times

please specify ?____________________________________________________________________

2. How many times, during the preceding year, have you visited a nurse (please circle the most appropriate alternative)

Due to diabetes 0 1-2 3-4 5-6 7-8 More than 8 times

Due to other reasons 0 1-2 3-4 5-6 7-8 More than 8 times

please specify ?____________________________________________________________________

3. How much does diabetes *per se* and/or its treatment disturb your normal life? (please circle one)

1 Not at all

2 Slightly

3 Moderately

4 Quite a lot

5 Very much

4. How much do the potential diabetes related complications disturb your normal life? (please circle one)

0 I do not have any complications

1 Not at all

2 Slightly

3 Moderately

4 Quite a lot

5 Very much

5. What is your last measured HbA_1c_ value? ____________%

6. What does this above-mentioned value tell about an average daily glycaemic control during the preceding 6-8 weeks? (please circle one)

1 That it is at a good level

2 That it is at a satisfactory level

3 That it is at a high level

7. Are you satisfied with your current HbA_1c_ level?

1 Yes

2 No

8. Are you satisfied with your current insulin regimen?

1 Yes

2 No

9. How often have you experienced perceptions of low blood glucose levels (perceptions of hypoglycaemia) during the past 4 weeks? (please circle one)

0 0 times

1 1-2 times

2 3-4 times

3 5-6 times

4 7-8 times

5 More than 8 times

10. How often, during the past 4 weeks, have you measured blood glucose values below 3 mmol/l without having felt that you have low blood glucose levels (e.g., asymptomatic low)? (please circle one)

0 0 times

1 1-2 times

2 3-4 times

3 5-6 times

4 7-8 times

5 More than 8 times

11. Are you afraid of hypoglycaemias?

1 Yes

2 No

12. Besides diabetes, do you suffer from any other chronic diseases?

1. No

2. Yes, which? _____________________________________________
